# Contrasting responses of above- and below-ground herbivore communities along elevation

**DOI:** 10.1007/s00442-020-04778-7

**Published:** 2020-10-19

**Authors:** Camille Pitteloud, Patrice Descombes, Sara Sànchez-Moreno, Alan Kergunteuil, Sébastien Ibanez, Sergio Rasmann, Loïc Pellissier

**Affiliations:** 1 Landscape Ecology, Department of Environmental Systems Science, ETH Zürich, Institute of Terrestrial Ecosystems, Universitätstrasse 16, 8092 Zürich, Switzerland; 2 Unit of Land Change Science, Swiss Federal Institute for Forest, Snow and Landscape Research WSL, 8903 Birmensdorf, Switzerland; 3 Department of Environment and Agronomy, National Institute of Agriculture and Food Research and Technology, 28040 Madrid, Spain; 4 Functional Ecology Laboratory, Institute of Biology, University of Neuchâtel, Rue Emile-Argand 11, 2000 Neuchâtel, Switzerland; 5 Laboratoire D’Écologie Alpine (LECA), UMR CNRS5553, Université de Savoie, 73376 Le Bourget-du-lac, France

**Keywords:** Environmental gradient, Species richness, Herbivory, Functional traits, Ecological interactions

## Abstract

Above- and below-ground herbivory are key ecosystem processes that can be substantially altered by environmental changes. However, direct comparisons of the coupled variations of above- and below-ground herbivore communities along elevation gradients remain sparse. Here, we studied the variation in assemblages of two dominant groups of herbivores, namely, aboveground orthoptera and belowground nematodes, in grasslands along six elevation gradients in the Swiss Alps. By examining variations of community properties of herbivores and their food plants along montane clines, we sought to determine whether the structure and functional properties of these taxonomic groups change with elevation. We found that orthoptera decreased in both species richness and abundance with elevation. In contrast with aboveground herbivores, the taxonomic richness and the total abundance of nematode did not covary with elevation. We further found a stronger shift in above- than below-ground functional properties along elevation, where the mandibular strength of orthoptera matched a shift in leaf toughness. Nematodes showed a weaker pattern of declined sedentary behavior and increased mobility with elevation. In contrast to the direct exposal of aboveground organisms to the surface climate, conditions may be buffered belowground, which together with the influence of edaphic factors on the biodiversity of soil biota, may explain the differences between elevational patterns of above- and below-ground communities. Our study emphasizes the necessity to consider both the above- and below-ground compartments to understand the impact of current and future climatic variation on ecosystems, from a functional perspective of species interactions.

**Electronic supplementary material:**

The online version of this article (10.1007/s00442-020-04778-7) contains supplementary material, which is available to authorized users.

## Introduction

The study of community properties along environmental gradients is a fundamental and necessary step toward better predictions of the functioning of natural systems (Garnier et al. 2016; Mayor et al. 2017). In most of the taxonomic groups and trophic levels studied so far, biodiversity shifts along elevation gradients have been generally associated with the variation in climate that exists between low- and highlands (Rahbek 1995; Hodkinson 2005; Mccain and Grytnes 2010; Sergio and Pedrini 2010; Guo et al. 2013). One of the most accurate climatic correlates of elevation is temperature, and the linear decrease of temperature with elevation represents a major constraint for species distribution along elevation gradients (Peters et al. 2016). Temperature may stimulate metabolic and diversification rates, fostering more species in warm environments (Clarke & Fraser 2004; Allen et al. 2006; Hatfield and Prueger 2015), while cold extremes may generate a stress effect, filtering the composition of communities that can occupy the alpine belt (Körner 2003; Sierra-Almeida et al. 2009; Hoiss et al. 2012; Buckley and Huey 2016). To date, our understanding of the variation of species assemblages along montane gradients is biased toward aboveground organisms (McKenzie et al. 2013), while the ecological signature of elevation on soil communities remains largely unknown (Martiny et al. 2006; Pellissier et al. 2014a).

Above- and below-ground communities both influence important ecological processes, including nutrient cycling and trophic interactions (Bardgett et al. 1998; Adams and Wall 2000; Wardle et al. 2004). Their common role in mediating some of the most important aspects of ecosystem functioning calls for more comprehensive studies on the coupled variation of both systems along environmental gradients. Climatic variation along elevation clines may have different effects on below- compared to above-ground organisms (Adams and Wall 2000) as surface conditions can be buffered in the soil compartment (Beyens et al. 2009), which may result in different species turnover rates between both systems along environmental gradients. For instance, a general decline in species richness along elevation or latitude has been documented for multiple aboveground taxonomic groups of plants and animals (Hodkinson 2005; Sharma et al. 2009; Mccain and Grytnes 2010; Guo et al. 2013; Descombes et al. 2017a, b). In contrast, the few studies conducted on soil systems thus far have not demonstrated a similar association between elevation and taxonomic richness and/or abundance (Margesin et al. 2009; Fierer et al. 2011; Jarvis et al. 2015; Kergunteuil et al. 2016; van den Hoogen et al. 2019) or even found an increase of these community indices with elevation. For instance, Pellissier et al. (2014a) reported a higher fungal richness and phylogenetic diversity at lower temperatures and higher moisture conditions. Similarly, Kergunteuil et al. (2016) found that the abundance and metabolic footprint of soil nematodes increased when moving from forested low-elevation sites up to Alpine grasslands. If such contrasting patterns of biodiversity generalize to others components of trophic networks, such as major herbivore groups, it would lead to a shift and decoupling of ecosystem processes, from above- to below-ground, depending on where the system is along the climatic gradient (Adams and Wall 2000; Hooper et al. 2000).

The species interactions that drive ecosystem processes are mediated by functional traits. Reciprocally, functional characteristics modulate ecological interactions and can be used together with community structure indices to understand community responses to environmental gradients (Diaz et al. 1998). The choice of relevant functional traits that mediate species–environment interactions through abiotic and biotic mechanisms presupposes a good knowledge of the taxa under study and their ecology (Mlambo 2014; Nock et al. 2016). Studies on the variation of functional traits with elevation are common for plant traits such as height (Moles et al. 2009), leaf structure (Reich et al. 1998), or leaf resource content (Shipley and Vu 2002). For insects, a decrease in body size, in wing length, or shifts in coloration associated with heat absorption have also been related to the elevation gradient (Hodkinson 2005). In contrast, the characterization of functional strategies for soil communities along elevation remains overly marginal (Kergunteuil et al. 2016), although some recent studies have attested to the functional response of soil organisms to the elevation gradient (Bond-Lamberty et al. 2016; Looby et al. 2016). Yet, as underground systems are less exposed to climatic conditions (Adams and Wall 2000), we expect that functional trait composition and species turnover along elevation is dictated by different structural forces than those acting on the surface.

Climatic variation shapes functional trait composition at the community level (Diaz et al. 1998), in turn, potentially modifying the mode and strength of species interactions (Hillyer and Silman 2010), and ultimately, ecosystem processes (Tylianakis et al. 2008). For instance, plant–herbivore interactions are modulated by the coupling of plant resistance and feeding-related traits (Moles et al. 2013). In this regard, shifts in the composition and functional identity of herbivores along elevation gradients (Hodkinson 2005) has been shown to reduce the intensity of herbivory (Rasmann et al. 2014), and should, therefore, change the investment of plant defences along elevation (Pellissier et al. 2012). As a consequence, variation in plant and herbivore functional traits along environmental gradients should modify species interactions within ecosystems. Consequently, studying both above- and below-ground community composition and functional traits could provide valuable information on the plant–herbivore relationships within the two sub-systems along elevation gradients and enable a stratified characterization of ecosystem functioning.

In this study, we explore structural properties and functional constituents of above- and below-ground herbivore communities along six elevational transects in the Swiss Alps. We compare the assemblage of two groups of herbivores: orthoptera that feed on leaves, and herbivore nematodes that feed on roots. Orthoptera is among the most influential aboveground insect herbivores in open habitats, removing high percentages of plant biomass in natural meadows (up to 30%, Blumer and Diemer 1996). Herbivory pressure exerted by orthoptera has been reported to decrease at high elevation, as species richness and abundance are reduced at that level (Scheidel and Bruelheide 2001; Hodkinson 2005; Descombes et al. 2017a). Nematodes are belowground organisms characterized by high functional, trophic, and taxonomic diversity. Herbivore nematodes are capable of substantial uptakes of plant root biomass (Ingham and Detling 1990; Hodda et al. 2009) and are distinguished into functional feeding groups including five plant root-feeding strategies (sedentary endoparasites, migratory endoparasites, semi-endoparasites, ectoparasites, and epidermal cells and root-hair feeders; Yeates et al. 1993). Responses of herbivore nematodes to elevation have seldom been described with reports of decrease (Dong et al. 2017) or increase (Kergunteuil et al. 2016) in taxonomic richness and abundance at a higher elevation. To date, concomitant investigations of both orthoptera and nematode communities, their functional responses and those of local plant communities along elevation, are still lacking. In this study, we address these questions with the following expectations:

1. Species richness and total abundance should decline more above- than below-ground along elevation because strong aboveground temperature shifts are buffered belowground. Differential rates of change in communities along elevation may lead to decoupled above- and below-ground community composition.
2. The functional traits of aboveground herbivore communities should vary more along elevation than the abundance of the functional feeding groups of belowground herbivores nematodes (e.g. epidermal/root hair feeders, ectoparasites) because of attenuated abiotic variations. In particular, we expect that feeding strategies, reflected by the above-mentioned functional properties, should be more stable among below- compared to above-ground herbivores.
3. Variations in herbivore traits should match those of plant traits along elevation. In particular, we expect the mandibular strength of orthoptera to increase toward high elevation in response to increasing plant leaf toughness in more stressful environmental conditions. We further expect different responses to the elevation between specialists (e.g. sedentary and migratory endoparasitic nematodes) and generalist feeders due to change in plant traits and edaphic conditions.

## Methods

### Study sites

To study above-belowground plant–herbivore interaction along elevation gradients, we selected six elevation transects spanning the major macro-climatic and environmental conditions (i.e., climate and bedrock type) of the Central Alps (see Fig. S1). We selected eight study sites per elevation transect in open, non-woody areas with elevations ranging from 578 m to 2417 m, and an average elevation difference between sites of 240 m. Study sites were located in semi-natural grasslands characterized by low impact from agricultural practices in land-use and pasture. Most of the low to medium elevation sites corresponded to dry meadows and pastures in the Swiss inventory for national protected areas (Federal Act on the Protection of Nature and Cultural Heritage (LPN), status as of 1 January 2017; Article 18. Protection of animal and plant species, https://www.admin.ch), while high elevation sites were situated in alpine meadows with no mowing and low grazing pressure. Herbivores and plant surveys took place during the summers of 2016 and 2017 within a square area of 10 m × 10 m. Study plots were positioned within the study zone to represent the dominant vegetation type of the surrounding natural environment and were set a minimum three meters away from forest edges when present. Plant, orthoptera and nematode herbivore inventories were compiled when communities reached maximal species richness and abundance, gradually surveying low to high elevation sites, between early June and the end of August.

### Herbivore surveys

Among orthoptera, the Caelifera suborder includes only strictly vegetarian species, while Ensifera are omnivorous, but largely feed on plant material (Ingrisch and Köhler 1998; Baur et al. 2006). We, therefore, included both suborders in the subsequent analyses. Orthoptera surveys were performed under optimal weather conditions for insect activity, between 10 a.m. and 5 p.m. on days with maximal sunshine levels. Species determination was done through visual and auditory identification using the reference work for Swiss Orthoptera (Baur et al. 2006). We estimated the abundance of each species following a “Z” sampling pattern across the 100 m^2^ study area by counting all adult orthoptera specimens that were visually detected without distinguishing sex. Nematode sampling consisted in a random sampling of 15–20 soil cores (2 cm diameter, 10–25 cm depth) within a 2 m × 2 m area located in the middle of the 100 m^2^ study area to obtain 1 kg of soil after the removal of all rock pieces greater than 2 cm in diameter. The bulk soil was then mixed homogenously, and a sample of 300 g was collected and stored at 4 °C before nematode extraction. Twenty-five grams of fresh soil were air-dried and used to calculate soil moisture. Two-hundred grams of fresh soil were used to extract nematodes from each sample using a modification of the sieving and Baermann funnel method (Barker 1985). Once extracted, all nematodes were counted under a binocular microscope. After counting, nematodes were concentrated and mounted alive in a slide and at least 150 nematodes were identified to the genus or family level according to Bongers (1989). Nematodes were identified at 200 × or 400 × under an inverted microscope, and nematode abundances expressed as no. of nematodes standardized to 100 g of dry soil.

### Plant surveys

The vegetation inventories were first conducted in a circular subplot of 9 m^2^ positioned in a floristically homogeneous area within the 100 m^2^ plot, across which we further searched for additional rare species. Plant species determination was done following Swiss Floras (Lauber et al. 2012; Eggenberg and Möhl 2013). We visually estimated the relative cover of each plant species according to a 9-level scale (< 0.25, 0.25–0.5, 0.5–1, 1–5, 5–15, 15–25, 25–50, 50–75 and > 75%). Low abundance classes (i.e. < 0.25 and 0.25–0.5) were used to accurately account for the difference in the relative abundance of rare plant species. The median values of these classes were used in all subsequent statistical analyses.

### Herbivore functional trait measurements

To measure orthoptera incisive strength, we collected insect specimens in falcon tubes that were killed with cold treatment at − 20 °C. Mandibular trait measurements were performed following the approach described by Ibanez et al. (2013), for 90% of the study species using three specimens of each species and sex. When a species was observed in more than one study site, collection points were selected within and between the study areas to cover the full extent of elevation and geographical range of each species. After the extraction of the left mandible, we took photographs of each mouthpiece in triplicate using a high-resolution measuring digital microscope (Leica DVM6, Leica Microsystems, GmbH, Wetzler, Germany), which, together with the high-resolution photo stacking option available in Leica Application Suite X (LAS X) and Leica Map Premium software (Leica Microsystems), maximizes photographic resolution to increase measurement accuracy. The mandibular incisive strength (F_I_) was obtained *F*_*I*_~*F*_A_*L*_*A*_/*L*_1_1/*R*_1_ using the formula, where *F*_A_ is a proxy for the mandibular section area, *L*_A_ is the adductor muscle lever, *L*_i_ is the incisive lever and *R*_1_ is the incisive region length (Ibanez et al. 2013). Values were measured on mandible photographs using the ImageJ image processing program (Rueden et al. 2017). For nematodes, we used the Nematode INdicator Joint Analysis system **(**NINJA, Sieriebriennikov et al. 2014) to assign feeding groups to each nematode taxon, and extracted only the genus corresponding to plant-parasitic nematodes. The functional classification within herbivore nematodes includes (a) epidermal/root hair feeders, which generally induce low phytopathological damage, (b) ectoparasites, which feed on superficial root tissues, (c) semi-endoparasites, which partially introduce their body into the root tissue, (d) migratory endoparasites, which migrate through the root tissue, and (d) sedentary endoparasites, which complete their life cycle inside roots, thus establishing a permanent feeding site and inducing the formation of root galls or cysts.

### Plant functional trait measurement

Leaf functional traits were collected for 79% of the plant species occurring in the plant surveys. We measured four leaf functional traits that were initially considered as relating to climatic disturbance, but could also confer resistance to herbivory since they reflect leaf toughness and resource acquisition: specific leaf area (SLA), leaf dry matter content (LDMC), punch strength, and carbon-to-nitrogen ratio (C/N). We sampled a minimum of three individuals per species and site, however, when the species occurred at more than one study site along the elevation gradient, we accounted for potential intra–species variation by increasing the number of replicates across different elevations to a maximum of twelve by multiples of three for each additional site. We selected well-developed and healthy leaves that were moisturized directly after collection and stored at 4 °C with additional moisture for a maximum of 24 h before trait measurement. Measurements of SLA (calculated as the area of a fresh leaf divided by the dry weight and expressed in mm^2^ mg^−1^) and LDMC (the ratio of the leaf dry mass to the water-saturated weight in mg g^−1^) were performed following standard procedures (Cornelissen et al. 2003; Vaieretti et al. 2007). Dry weight was measured after oven-drying the fresh leaves at 55 °C for a minimum of 72 h. The leaf force to punch, which is considered to be the trait that best captures leaves’ mechanical properties that are relevant for herbivory (Sanson et al. 2001; Ibanez et al. 2013), was measured using a digital force gauge that records the force required to pierce the leaf lamina (IMADA CO., LTD. Toyohashi, Japan). Measurements were taken on fresh leaves with the measurement point selected to avoid leaf veins. Values are expressed in MN m^−2^ and were corrected for leaves with widths of less than the diameter of the gauge pin (2 mm). Leaf width was measured using a digital caliper gauge (0.01 mm precision). Total organic carbon (*C*) and nitrogen (*N*) amounts were determined for 89% of all species by dry combustion of ground leaf material using a CN elemental analyzer (NC-2500 from CE Instruments, Wigan, Lancashire, United Kingdom).

### Taxonomic richness and abundance along elevation

Species richness of plants and orthoptera insects, and the genera richness of herbivore nematodes were calculated as the sum of the number of taxa identified at each study site. Abundance was estimated using the total specimen count at each site for the orthoptera and no. of individuals per 100 g of dry soil for nematodes assemblages. The abundance of nematodes for one particular site that differed from others by a particularly thick layer of dead plant organic matter exceeded the median value of the total number of nematodes by a factor of ten. This likely indicates a bias in the sampling process such as the accidental inclusion of soil containing a non-representative high level of plant organic matter resulting in the extremely high value of nematodes abundance. This site was, therefore, removed from subsequent analyses. Relationships between elevation and response variables were tested using regression models that included the transect identity as a random factor using functions within the lme4 and lmerTest *R* packages (Bates 2008, Kuznetsova et al. 2017). All analyses were conducted using R (R Core Team 2019). The variation of orthoptera and nematode taxonomic richness and orthoptera abundance along elevation was tested elevation using generalized linear mixed-effects models for the Poisson distribution of count data (glmer) and we use a second-degree polynomial regression when the shape of the relationship was not linear. We also tested the variation of species richness and abundance along the elevation for both orthoptera suborders independently using the same model types. The nAGQ parameter was set to 0 for orthoptera and nematode models to ensure model convergence. The variation of the plant taxonomic richness was tested using a second-degree polynomial regression in a generalized linear model with a negative binomial family to avoid overdispersion of the residuals. We related nematode abundance data to elevation using a linear mixed effect model for Gaussian distribution data with a logarithmic transformation (log + 1). Overdispersion of the residuals of the models was verified by simulating standardized residuals from the fitted models in DHARMa R package (Hartig 2020).

### Co-inertia analyses and temperature variation along elevation

We conducted a co-inertia analysis to test the coupling of above- and below-ground herbivore communities. For each transect, we first performed a PCA on abundance matrices of orthoptera and nematodes independently using the function “dudi.pca” in the ade4 package (Thioulouse et al. 2018). We then used the first two factorial axes of the PCAs to apply the co-inertia criterion procedure and quantify the co-variance between the two tables (co-inertia function) using the RV coefficient. Values close to one indicate maximal co-variance between matrices. To further investigate how surface climate might be buffered in the soil, we compared the variation of above- *vs.* below-ground temperature along the elevation using soil temperature measured with data loggers (DS1921G-F5 HomeChip, Farnell, Zug, Switzerland) from October 2017 to September 2018 and air temperature data supplied by MeteoSwiss (2 m aboveground; 2020, Swiss Federal Office of Meteorology and Climatology).

### Functional traits along elevation

The functional classification of herbivore nematodes was used to reflect the functional aspects of belowground communities. We summed the abundance of each herbivore group to quantify their abundance at each site and performed a logarithmic transformation of the data (log + 1) to fulfil model assumptions. The mean community values (CM) of functional traits for orthoptera (i.e., incisive mandibular strength) and plants (i.e., SLA, LDMC, punch strength, C/N) were obtained by averaging the sum of the trait values by the total number of species. The community weighted means (CWM) accounting for species abundance were computed for the same traits and each site using where $R$ is the number of species, $Pi$ is the relative abundance of the species $i$, and $ti$ is the mean trait value of the species $i$. While the two metrics are based on means, CM reflects the average of life-strategies occurring within a community weighting all species equally, and CWM accentuates the ecological role of dominant species (Garnier et al. 2004). We used linear mixed-effects models with Gaussian data distributions to test whether abundances of the nematode functional groups were associated with the elevation gradient. For the variation of orthopteran functional trait metrics along elevation (i.e., CM and CWM), we fitted linear mixed-effects models for Gaussian data distribution. The weighted and unweighted CM of plant functional traits along the elevation were analyzed using the same models. We excluded the three lowest elevation sites of the Salgesch transect since they were more similar to steppic environments, with plant traits strongly biased toward functional responses that are typical of extremely dry environmental conditions (Volaire 2008; Delarze et al. 2015).

## Results

### Taxonomic richness and abundance along elevation

**Fig. 1:**
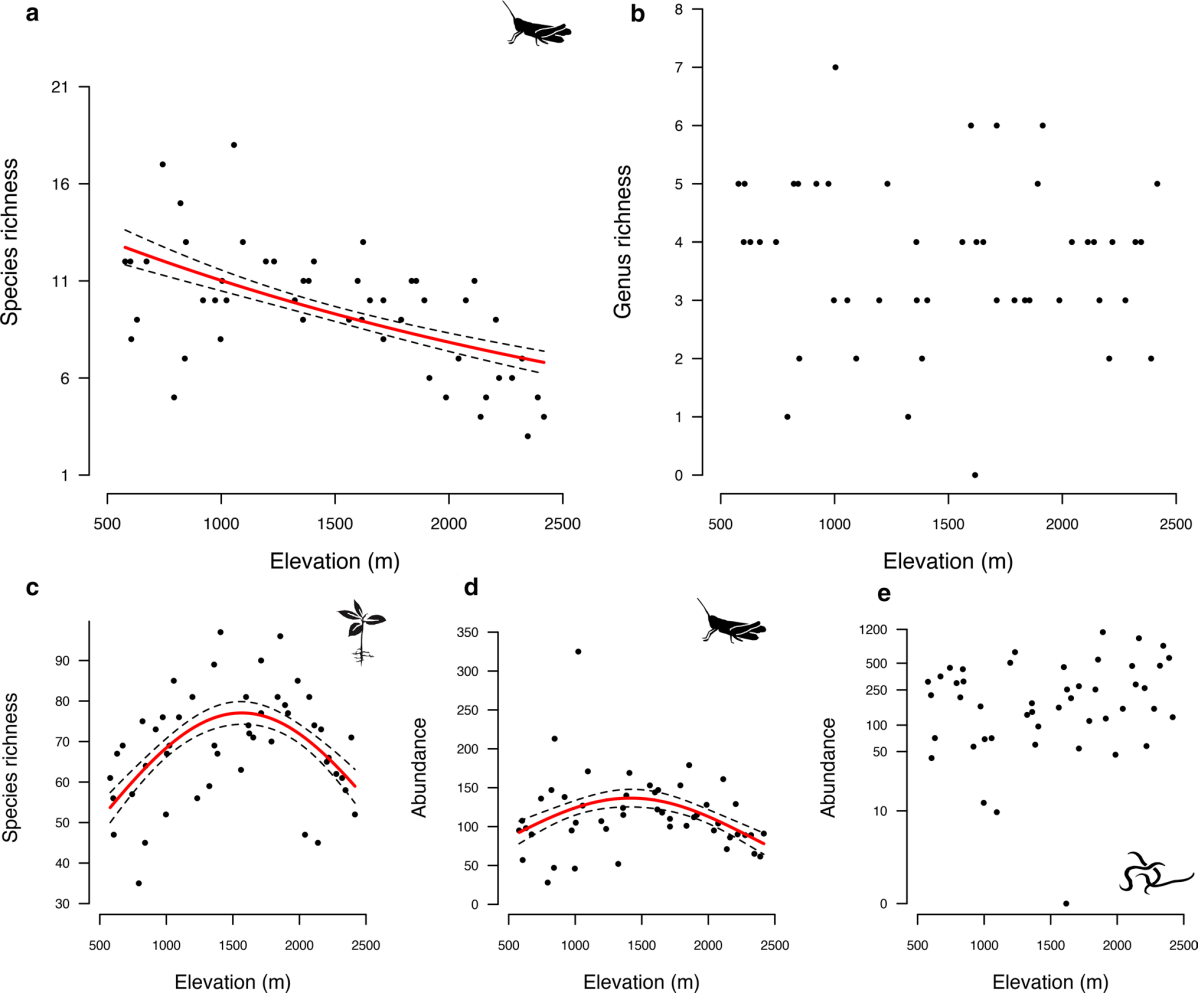
Illustration of the relationships between the elevation and community structure indices using linear and generalized linear mixed effects models with the taxonomic richness of (**a**) orthoptera, (**b**) herbivore nematodes, and (**c**) plants, and the specimen abundance of (**d**) orthoptera communities and (**e**) herbivore nematodes (log-transformed). Regression lines of fitted values and standard error intervals are only displayed for relationships that are statistically significant. A significant decrease in taxonomic richness was found for orthopteran and plant but not for herbivore nematode communities. The abundance of orthoptera also decreased with the elevation, a signal that was not retrieved for herbivore nematodes

From vegetation surveys, we identified 526 plant species belonging to 251 genera and 69 families. From orthoptera surveys, 48 species including 19 Ensifera and 29 Caelifera taxa were determined. In total, we identified 55 nematode genera, comprising 14 herbivore genera that corresponded to five different plant-parasitic types (i.e., ectoparasites, epidermal/root hair feeders, semi-endoparasites, migratory, and sedentary endoparasites). The nematode taxa identified, their functional classification, and percentage contribution of herbivore functional groups to the nematode community are indicated in Table S1. We found that the species richness of orthoptera and plant significantly decreased with elevation following linear and hump-shaped relationships, respectively (Fig. 1a, c, Table 1). When tested independently for Caelifera and Ensifera, the same declining trends were found, except for the variation of Caelifera species richness along elevation (Fig. S2, Table S2). This result contrasts with the nematode distribution patterns that presented no variation along the transects for the genus richness and the abundance (Fig. 1b, e, Table 1). Orthoptera abundance displayed a significant polynomial relationship with elevation, with the highest values found at mid-elevation (Fig. 1d, Table 1).

**Table 1:** Slope coefficients (slope estimate), *P* values and standard errors (SE) for linear and generalized linear mixed effects models testing the relationships between elevation and community indices

	Linear model			2nd degree polynomial model		
	Slope Estimate	*P* value	SE	Slope Estimate	*P* value	SE
Species richness						
Orthoptera	− 0.0003	< 0.001	0.0001			
Herbivore nematodes	− 0.0001	0.584	0.0001			
Plants	0.240	0.16	0.17	− 0.696	< 0.001	0.169
Abundance						
Orthoptera	− 0.273	0.006	0.10	− 1.053	< 0.001	0.099
Herbivore nematodes	0.0004	0.245	0.0003			

Values are provided for linear and 2nd degree polynomial models (when applicable) that included the transect identity as a random factor to quantify the variation of the species richness of orthoptera, herbivore nematodes, plants, and the species abundance of orthoptera and herbivore nematodes along the elevation. Overdispersion of the residuals was verified by simulating standardized residuals from the fitted models in DHARMa R package (Hartig 2020)

### Co-inertia analyses and temperature variation along elevation

Along the co-inertia analysis performed on each transect individually, the PCAs’ first two axes together explained more variance in nematode abundance than orthoptera (Table 3), accounting, on average, for 41% and 27% of the variance in orthoptera, and 79% and 15% in nematodes, respectively. The analyses suggested a partial decoupling between above- and below-ground herbivore communities with RV coefficients ranging from 0.20 to 0.44 (see Table 3). When comparing above *vs.* below-ground temperature along the elevation, we found that the monthly average and minimum temperature are generally higher belowground than at the surface (Fig. S3a & S3b) and that the temperature variance considerably decreased in the soil (Fig. S3c).

### Orthoptera and nematode traits along elevation

**Fig. 2:**
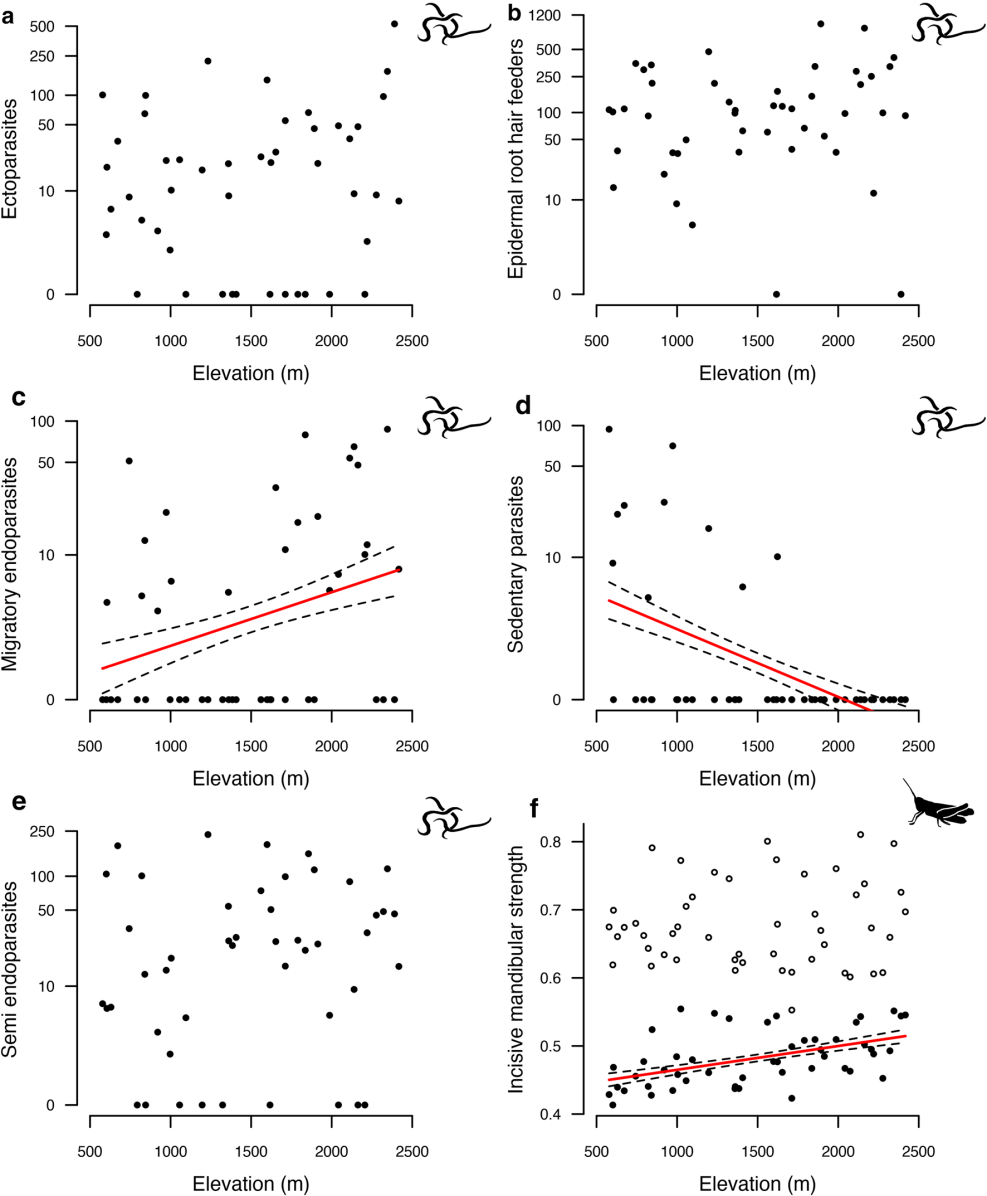
Illustration of the relationships between elevation and the abundance of nematode functional groups of (**a**) ectoparasites, (**b**) epidermal root feeders, (**c**) migratory endoparasite, (**d**) sedentary endoparasites, (**e**) semi-endoparasites, and (**f**) the CM of orthoptera incisive mandibular strength using linear mixed effects models for Gaussian distribution data. Nematodes abundance data were log-transformed. Females orthoptera are represented by white circles and males by black circles. The regression line of the fitted values and the standard error intervals are displayed only for significant relationships. The abundance of nematode functional groups was generally steady or increased along the elevation, except for the sedentary endoparasites that showed a decrease toward high elevation. A positive relationship was found between the incisive mandibular strength of males orthoptera along the same gradient

Among the five herbivore nematode functional groups, we only found that sedentary parasites decreased significantly in abundance with elevation (Fig. 2d, Table 2). While no clear trend in abundance variation was visible for ectoparasites, epidermal/root hair feeders, or semi-endoparasites (Fig. 2a, b, e, Table 2), the abundance of migratory endoparasites was significantly higher at high elevation (Fig. 2c, Table 2). The CM of the incisive mandibular strength of orthoptera increased with elevation for males, while for females, values were stable along the gradient (Fig. 2f, Table 2) and largely outreached those of males. The CWM of this trait followed the same pattern (Fig. S4, Table S3).

**Table 2:** Slope coefficients (slope estimate) and* P* values and standard errors (SE) for linear mixed effects models testing the relationships between the elevation and the abundance of herbivore nematode functional groups using a logarithm transformation, male and female orthoptera incisive mandibular strength, and plant functional traits

	Slope Estimate	*P* value	SE
Abundance of nematode functional groups			
Ectoparasites	0.0002	0.56	0.0004
Epidermal root hair feeders	0.0002	0.66	0.0003
Migratory endoparasites	0.001	< 0.05	0.0004
Sedentary parasites	− 0.001	< 0.001	0.0003
Semi-endoparasites	0.0004	0.32	0.0004
Orthoptera mandibular strength			
Male	0.00003	< 0.001	0.00001
Female	0.00001	0.55	0.00002
Plant functional traits			
SLA	− 0.001	0.01	0.0003
LDMC	− 0.01	< 0.01	0.003
Punch strength	0.0002	< 0.001	0.00004
C/N	0.001	< 0.001	0.0004

The abundance of the functional groups of belowground herbivore nematodes displayed weaker variation with elevation than the functional traits of aboveground herbivore communities

### Plant traits along elevation

**Fig. 3:**
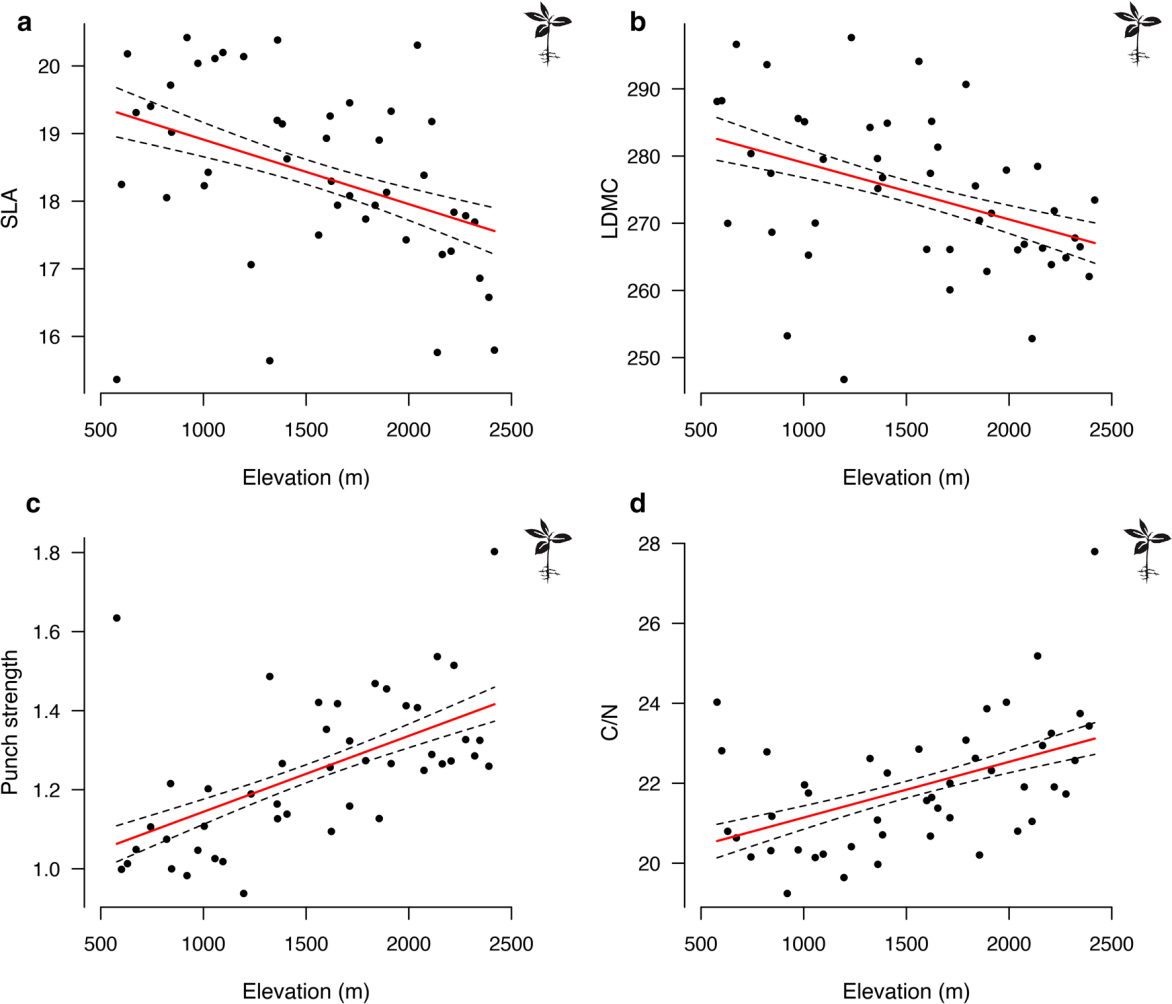
Relationships between plant functional trait community means (CM) and elevation using linear mixed effects models for (**a**) SLA, (**b**) LDMC, (**c**) punch strength, and (**d**) C/N. Regression lines of fitted values and standard error intervals are only shown for significant relationships. All the tested plant functional traits displayed a significant relationship with the elevation, that was positive for the SLA and LDMC and negative for the C/N and the punch strength

The CM of SLA and LDMC showed a significant negative relationship with elevation (Fig. 3a, b, Table 2), whereas a positive relationship was found for punch strength and C/N (Fig. 3c, d, Table 2), the latter being concurrently explained by a decrease in nitrogen and an increase in carbon. The results of the regression models applied to CWM generally indicate similar trends to those found for CM (Fig. S5, Table S3).

## Discussion

Variations in above- and below-ground herbivore communities along elevation gradients have rarely been directly compared to date. Here, we have focused on two dominant groups of herbivores in grasslands, aboveground orthoptera and belowground nematodes, and studied variation in assemblages along six elevation gradients in the Swiss Alps. We examined variations in community taxonomic properties and the functional traits of above- and below-ground herbivores, and their food plants along montane gradients. We have shown that variations in community indices along elevation considerably differ between above- and below-ground organisms. The evenness of nematode taxonomic richness along the elevation gradient contrasted with the decrease in orthoptera species richness at the highest elevation and may be related to the tempering effect of stressful abiotic conditions that limits environmental filtering in the soils. The response of above- and below-ground communities to elevation also differed as regard to abundance. We found no relationship for nematodes contrasting with an upward decline of orthoptera along the gradient. We have also found a stronger shift in above- than below-ground functional properties along elevation. The mandibular strength of orthoptera matched a shift in leaf punch strength along elevation, while nematodes showed a pattern of elevational variation only through a decline in sedentary endoparasites and an increase of migratory endoparasites with elevation. We discuss our results in terms of abiotic conditions that act above- and below-ground, and examine their implications for shifts in plant–insect herbivore interactions along elevation gradients.

The species richness and abundance of orthoptera decreased with elevation (Fig. 1a, d), while for nematodes, no variation was observed (Fig. 1b, e). A decrease in aboveground biodiversity is generally associated with a shift in climatic conditions along elevation although such strong climatic gradients may not extend to belowground communities (Bryant et al. 2008; Fierer et al. 2011). If environmental conditions apply similarly above- and below-ground, we should find an equivalent variation in taxonomic richness and abundance for above- and below-ground assemblages. Contrary to this expectation, the co-inertia analyses indicated generally low congruence between orthoptera and nematode communities (Table 3), suggesting that the environmental gradients shaping soil communities are different from those acting on the surface. Among the environmental conditions that shift strongly with elevation, the temperature gradient and in particular, thermal variability may be buffered for soil biota (Fig. S3), differentially shaping the structure of above-compared to below-ground communities (Beyens et al. 2009; Ryalls et al. 2013). We found a monotonical decrease in orthoptera species richness that directly follows the gradual decrease in temperature with elevation (Barry 2008), while orthoptera abundance peaks at mid-elevation, where plant species richness is highest (Fig. 1c; Haddad et al. 2001; Descombes et al. 2017b). In contrast, the taxonomic richness and the abundance of nematodes did not vary with elevation (Fig. 1b, e). This may indicate the limited influence of temperature decline and variability along the elevation gradient on soil communities. It also suggests that edaphic factors (e.g. soil fertility and humidity) may vary along montane clines to shape soil communities, which, together with buffered surface conditions, may result in patterns that strongly diverge from those observed aboveground (Kergunteuil et al. 2016; van den Hoogen et al. 2019). In agreement with Kergunteuil et al. (2016) who analyzed nematode community properties along elevation transects, our study found that species diversity and abundance were higher in alpine meadows. Hence, indices of community structure that respond to elevation should fundamentally differ depending on whether the study compartment is located above or below-ground. These results also parallel biodiversity patterns occurring at a wider biogeographic scale. The distribution of orthopteran diversity was found to be higher in Southern Europe (Hochkirch et al. 2016) whilst the abundance and diversity of belowground biota were shown to increase in temperate regions (van den Hoogen et al. 2019). Alongside variation in taxonomic richness and abundance, we also expected a weaker variation of functional properties in above- compared to below-ground communities.

**Table 3:** Results of PCAs and the co-inertia analyses. For each transect are given the percentage of variance explained by the first two axes of the PCAs applied to orthoptera and nematode abundance matrices and RV coefficients of the co-inertia analyses performed on PCA

	Orthoptera		Nematodes		RV coefficient
	Axis 1	Axis 2	Axis 1	Axis 2	
Transect					
Bex	30.7	29.7	87.4	8.7	0.35
Calanda	55.3	21.7	93.8	5.1	0.25
Salgesch	38.8	32.6	75.3	24.4	0.20
Grindelwald	44.7	26.5	69.4	18.0	0.20
Martigny	37.3	25.6	54.9	29.6	0.44
Faido	38.6	24.5	94.6	4.2	0.25

The co-inertia analyses indicated a partial decoupling between above- and below-ground herbivore abundance matrices suggesting that the surface climatic conditions might be buffered belowground where edaphic factors govern to shape soil communities

Both orthoptera and nematode communities showed some degree of functional changes with elevation. The CM of mandibular strength for male orthoptera increased with increasing elevation, while the community values for females were steady and systematically greater. Morphological differences between sexes have been documented for this insect group (Laiolo et al. 2013), and we showed here that sexes responded differently to elevation. Because mandibular strength influences plant ingestion, higher values found for females could underscore the greater importance for this sex to bypass plant mechanical barriers at any elevation. This would ensure a nutrient intake that is capital for the production of eggs (Hochkirch and Gröning 2008). For males, although a minimum nutrient intake is necessary to guarantee survival, they may invest less in their feeding ability. Hence, the constant and high mandibular strength of females, and the increase for males with elevation possibly represents a response to plant physical resistance at high elevation where greater mandibular strength would help the ingesting of tougher plants (Ibanez et al. 2013). With regards to nematodes, we found a significant increase in abundance for migratory endoparasitic nematodes and a significant decrease for sedentary endoparasites, with no variation along elevation for nematodes characterized as ectoparasites, semi-endoparasites, or epidermal/root hair feeders. Both sedentary (*Meloidogyne*, *Heterodera*) and migratory (*Pratylenchus*) endoparasitic nematodes are able to feed on large numbers of plant species, being extremely polyphagous (Jones and Fosu-Nyarko 2014; Truong et al. 2015). Plant-parasitic nematodes are seldom studied in natural systems, and little information is available on the effects of elevation on parasitic nematodes. The limited information available, however, reported increased *Pratylenchus* and low *Meloidogyne* abundances at high elevations in tropical areas (Fogain 2001; Gaidashova et al. 2009; Avelino et al. 2009; Kamira et al. 2013). Our results showed that low-specialized belowground herbivory is equally distributed along elevation transects, while the most specialized herbivore conditions, represented by sedentary and migratory endoparasitism (Perrine-Walker 2019), present peak abundances at different elevations, respectively at 578 m and 2346 m. Competition among *Pratylenchus* and *Meloidogyne* has been detected in crops under experimental and natural conditions (Avelino et al. 2009; Fontana et al. 2015), and similar processes have been described in natural systems between *Pratylenchus* and other endoparasitic nematodes such as *Heterodera* (Brinkman et al. 2005). Besides plant functional traits and other ecological factors, such competition may play a role in structuring herbivore nematode communities across environmental gradients.

If functional traits govern the trophic interplay between plants and herbivores, and reflect community responses to environmental changes, they also reshape plant–insect herbivore interactions along elevation gradients. Influenced or not by abiotic constraints, the abundance of herbivores is also involved in modulating the functional interface between herbivore feeding abilities and plant defence (War et al. 2012). We have investigated a set of plant functional traits that represent physical resistance against herbivory. Among the four plant functional traits studied, we found that the CM of SLA and LDMC decreased with increasing elevation, while punch strength and the C/N ratio showed opposite trends. The increase in C/N and punch strength are indicators of a greater physical resistance of alpine plant communities. This is in line with the review of Moreira et al. (2018), who concluded that plants adapted to stressful environmental conditions tend to invest more in constitutive defence, which include some chemical but mostly physical defences. Changes in plant leaf traits correspond to the increase in mandibular strength in male orthoptera with increasing elevation. However, we lack data on root properties to properly assess changes in nematode communities. Due to the technical difficulties involved in measuring root functional traits, the imprint of herbivore abundance on root defence is relatively unexplored, with only a few studies focusing on chemical responses to root herbivory (Kaplan et al. 2008; Rasmann et al. 2011). Without knowledge of root defensive traits against herbivory, it remains difficult to connect nematodes and plant functional responses. However, since root biomass generally increases with elevation (WeiLing et al. 2010), plants may support constant herbivory pressures along elevation gradients based on tolerance rather than defensive response. Although this requires further analyses aiming at tracking different defence/tolerance trade-offs in above- and below-ground compartments, our study showed that at the surface, herbivore abundance declines with elevation while no variation or opposite patterns are observed in the soil. Our study also found that herbivore abundance matrices are partially decoupled and that the variation of functional responses is of greater amplitude for aboveground communities. These findings suggest that the plant–insect herbivore relationships in aboveground systems, in contrast to those belowground, are controlled by a set of abiotic and biotic forces that are unique to the study compartment, and should be studied accordingly. The decline in herbivore abundance along the elevation gradient documented for aboveground insect herbivores (Reynolds and Crossley 1997; Pellissier et al. 2014b; Descombes et al. 2017a) may not exist belowground. As a result of the increase in nematode abundance along elevation gradients, plant defences in roots may not show the same decline as documented for leaves (Pellissier et al. 2012; Callis-Duehl et al. 2017). We, therefore, propose that plant defence and herbivory relationships of above- vs. below-ground compartments react differently to environmental change, which calls for a greater effort to document belowground plant–herbivore interactions. However, the factors that operate in the structuring of plant–herbivore interactions remain unexplored, particularly for soil systems. Given the specificities of above- and below-ground systems, we believe that a line of research that considers both community types, in a functional and network perspective, is required to identify the drivers of species interaction, and to anticipate how climate change will affect distinct ecosystem compartments and functioning.

## Electronic supplementary material

Below is the link to the electronic supplementary material.

Supplementary file1 (DOCX 41672 kb)
