# Supplementary material for: Contrasting responses of above- and below-ground herbivore communities along elevation
Source: Oecologia. 2020 Oct 19;194(3):515–28. doi: 10.1007/s00442-020-04778-7 (PMC7644536; doi:10.1007/s00442-020-04778-7)
Supplement: Supplementary file 1 — Supplementary file1 (DOCX 41672 kb) [file 442_2020_4778_MOESM1_ESM.docx]

**Contrasting responses of above- and below-ground herbivore communities along elevation**

Camille Pitteloud^1,2^, Patrice Descombes^2^, Sara Sànchez-Moreno^3,^ Alan Kergunteuil^4^, Sébastien Ibanez^5^, Sergio Rasmann^4^, Loïc Pellissier^1,2^

^1^ Landscape Ecology, Institute of Terrestrial Ecosystems, Department of Environmental Systems Science, ETH Zürich, 8092 Zürich, Switzerland

^2^ Unit of Land Change Science, Swiss Federal Institute for Forest, Snow and Landscape Research WSL, 8903 Birmensdorf, Switzerland

^3^ National Institute of Agriculture and Food Research and Technology, Department of Environment and Agronomy, 28040 Madrid, Spain

^4^ Functional Ecology Laboratory, Institute of Biology, University of Neuchâtel, 2000 Neuchâtel, Switzerland

^5^ Laboratoire d’Écologie Alpine (LECA), UMR CNRS 5553, Université de Savoie, 73376 Le Bourget-du-lac, France

**Electronic supplementary material**

**Supplementary figures**

**Fig. S1** Map illustrating the location of the different study transects across the Swiss Alps

**Fig. S2** Illustrations of the relationships between elevation and species richness and abundance tested independently for (a, c) Caelifera and (b, d) Ensifera suborders using generalized linear mixed effects models. Regression lines of fitted values and standard error intervals are only shown for significant relationships.

**Fig. S3** Plot of the elevation against (a) the monthly temperature average, (b) the monthly temperature minimum, (c) the monthly temperature variance for above (red shades) and belowground (blue shades) measurements, with darker and lighter colors representing respectively the winter (and summer periods. Belowground temperature data were collected along each transect for half of the study sites using temperature loggers (DS1921G-F5 HomeChip, Farnell, Zug, Switzerland) parametrized at a sampling rate of 240 minutes with a resolution of 0.5°C. The loggers were wrapped in parafilm, protected by a silicone capsule and buried 4 cm deep in the ground from October 2017 to September 2018. Air temperature data measured 2 m above the ground were supplied by MeteoSwiss (2020, Swiss Federal Office of Meteorology and Climatology) for the same period, from meteorological stations located in the south, north, west and east central Swiss Alps (n=179), ranging from 600 m to 2,400 m.

**
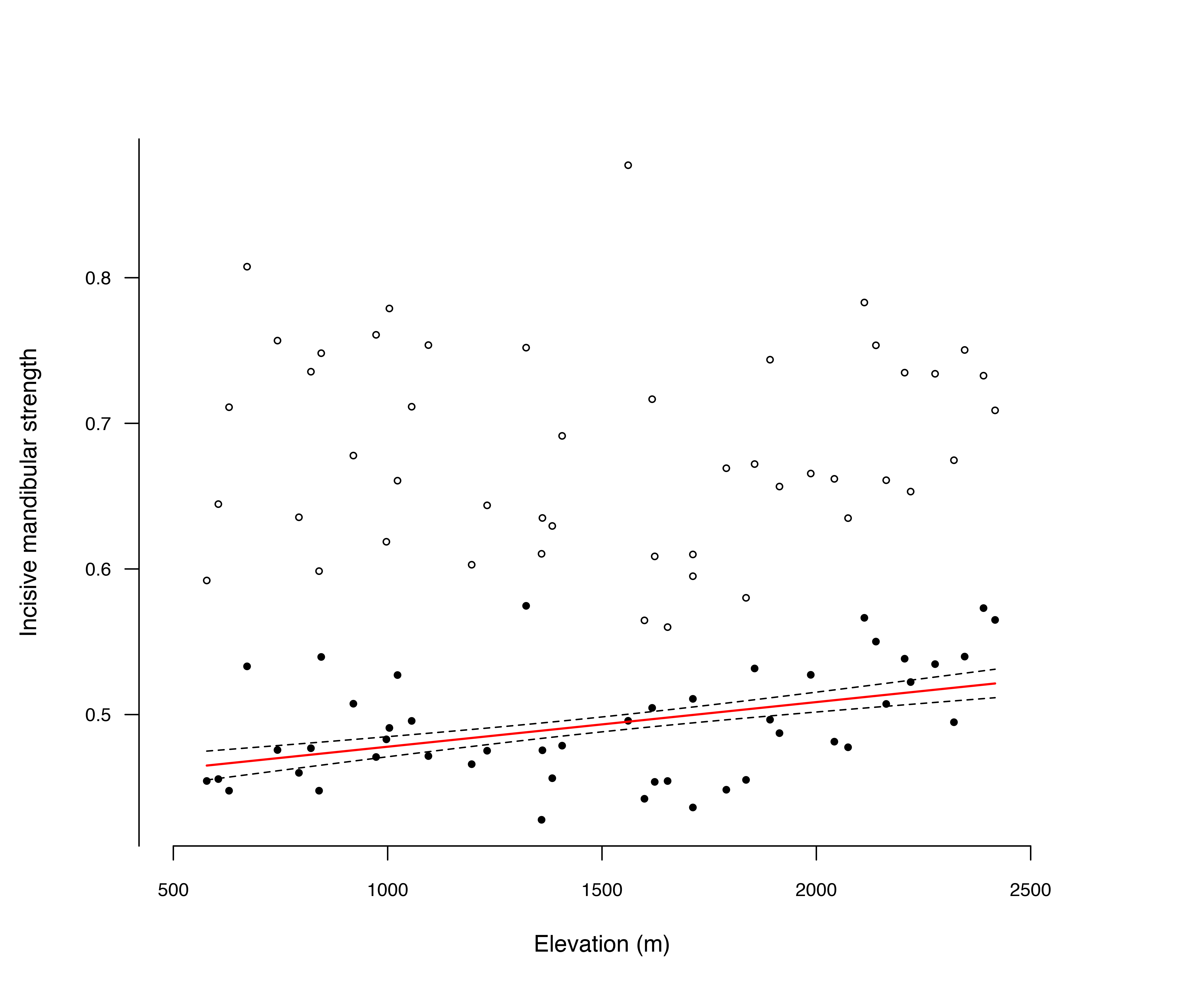
**

**Fig. S4** Variation in orthoptera incisive mandibular strength CWM with elevation obtained from a linear mixed effects model. The significant increase in CWM for males along the elevation gradient, and the steadier relationship found for females are similar to the results obtained through CM calculations. Regression lines of fitted values and standard error intervals are only shown for significant relationships.

**Fig. S5** Variation in the plant functional traits (CWM) with elevation obtained from a linear mixed effects model for (a) SLA, (b) LDMC, (c) punch, and (d) C/N. While only the CWM of SLA was found to significantly decrease with elevation, trends for other traits can be compared to the relationships found for CM calculations. Regression lines of fitted values and standard error intervals are only shown for significant relationships.

**Supplementary tables**

**Table S1** Mean (± SD) total number of nematodes per transect (including all trophic groups). The percentage of total herbivores and the percentage of different herbivore functional groups are indicated. *Axonchium*, *Gracilacus*, *Longidorella*, *Paratylenchus*, *Trophurus* and *Tylenchorhynchus* were classified as ectoparasites. *Helicotylenchus*, Hoplolaimidae, *Pararotylenchus*, and *Rotylenchus* were classified as semi-endoparasites. *Heterodera* and *Meloidogyne* were considered sedentary parasites. *Pratylenchus* was classified as a migratory endoparasite and Tylenchidae as an epidermal/root hair feeder.

| Transect | Bex | Calanda | Faido | Gindelwald | Martigny | Salgesch |
| --- | --- | --- | --- | --- | --- | --- |
| Mean total number of nematodes | 678.8 | 1861.4 | 1221.9 | 660.2 | 2056.1 | 849.7 |
| ± SD | ±302.3 | ±2423.8 | ±853.7 | ±368.2 | ±1936.3 | ±738.3 |
| Herbivores (%) | 26.3 | 20.7 | 31.4 | 25.1 | 31.9 | 28 |
| Sedentary endoparasites (% of herbivores) | 1 | 4.7 | 4.2 | 4.6 | 4.3 | 0 |
| Migratory endoparasites (% of herbivores) | 6.6 | 4.8 | 4.9 | 10.1 | 2.7 | 4.2 |
| Semi-endoparasites (% of herbivores) | 23.2 | 24.1 | 16.2 | 38.5 | 17.7 | 8 |
| Ectoparasites (% of herbivores) | 5.7 | 7.3 | 12.8 | 9.7 | 23.5 | 31 |
| Epidermal/root hair feeders (% of herbivores) | 63.4 | 59.1 | 61.9 | 37.1 | 51.7 | 56.7 |

**Table S2** Slope coefficients (slope estimate) and the *P* values of the generalized linear mixed effects models testing the relationship of species richness and abundance with elevation for Caelifera and Ensifera suborders.

|  |  | **Linear model** | | | **2nd degree polynomial model** | | |
| --- | --- | --- | --- | --- | --- | --- | --- |
|  |  | **Slope Estimate** | ***P* value** | **Slope Estimate** | | ***P* value** |  |
| **Species richness** | Caelifera | -0.0002 | 0.11 |  | |  |  |
|  | Ensifera | -0.001 | <0.001 |  | |  |  |
| **Abundance** | Caelifera | 0.104 | 0.34 | -0.891 | | <0.001 |  |
|  | Ensifera | -0.0004 | <0.001 |  | |  |  |
|  |  |  |  |  | |  |  |

**Table S3** Slope coefficients (slope estimate) and the *P* values of the linear mixed effects models testing the relationship between elevation and CWM of functional traits with respect to orthoptera mandibular strength with values given for males and females and plant SLA, LDMC, punch strength, and C/N leaf traits.

|  | **Slope Estimate** | ***P* value** |
| --- | --- | --- |
|  |  |  |
| **Orthoptera mandibular strength** |  |  |
| Males | 0.00003 | <0.01 |
| Females | 0.000003 | 0.88 |
| **Plant functional trait** |  |  |
| SLA | -0.003 | <0.001 |
| LDMC | -0.01 | 0.25 |
| Punch strength | 0.0002 | 0.38 |
| C/N | 0.001 | 0.06 |
